# Characterization of dendritic cells and follicular dendritic cells in the hepatic lymph nodes and liver of sheep experimentally infected with *Fasciola hepatica*

**DOI:** 10.1186/s13567-020-00757-1

**Published:** 2020-03-04

**Authors:** María Teresa Ruiz-Campillo, Verónica Molina-Hernández, María José Bautista, Isabel L. Pacheco, Rafael Zafra, Leandro Buffoni, Francisco Javier Martínez-Moreno, Alvaro Martínez-Moreno, José Pérez

**Affiliations:** 1 Department of Anatomy and Comparative Pathology, Faculty of Veterinary Medicine, University of Córdoba, Sanidad Animal Building, Rabanales Campus, Córdoba, Spain; 2 Department of Animal Health (Parasitology), Faculty of Veterinary Medicine, University of Córdoba, Sanidad Animal Building, Rabanales Campus, Córdoba, Spain

## Abstract

*Fasciola hepatica* has been shown to have a high capacity for immunomodulation of the host response, making the development of protective vaccines extremely difficult. One of these immunomodulation mechanisms is the impairment of dendritic cells (DC) maturation and, therefore, suppression of antigenic presentation. The aim of this study was to evaluate the pathological changes as well as the characterization of two antigen presenting cells, DC (CD1b, CD83 and MHC-II positive) and follicular dendritic cells (FDC) (CNA.42, S100 and CD83 positive) by immunohistochemistry in the hepatic lymph nodes (HLN) and livers of sheep during the early stages of infection with *F. hepatica* [9 and 18 days post-infection (dpi)], compared with an uninfected group (UC) as a control. The results revealed a marked hyperplasia of HLN germinal centres at 9 and, in particular, 18 dpi, with respect to the UC group, with coincidental increased expression of CNA.42 in FDC of lymphoid follicles and CD1b in the DC of paracortical areas at 18 dpi. However, the expression of MHC-II and CD83 decreased at 9 and, particularly, at 18 dpi in HLN compared with that in the UC group. Since both markers are related to active presentation of antigens by DC and FDC, the results of the present study suggest that, despite the marked hyperplasia of HLN and increase in DC and FDC numbers during early stages of infection, the DC and FDC antigenic presentation capacity, as suggested by the expression of the markers MHC-II and CD83, is suppressed by the parasite. This suppression was not observed in the liver, probably because of the low number of DC. This is the first study of the immunophenotype of DCs and FDC in sheep infected with *F. hepatica*.

## Introduction

Fasciolosis, caused by *F. hepatica*, results in major economic losses to the agricultural sector [1]. The parasite infects a wide range of domestic animals, including cattle, sheep and goats, and the disease has been recognized as an important zoonosis in Africa, Asia, Europe, America and Oceania [2, 3]. Control of the disease has traditionally been based on the use of anthelmintic drugs such as triclabendazole. Nevertheless, resistance to this and other drugs, together with public concern about the presence of drug metabolites in foodstuff, is increasing in numerous countries [4]. Because of this, there is increasing interest in the development of an immunological method of control for the disease [5, 6]. Despite major efforts during the last two decades, the search for an effective vaccine to control fasciolosis has been slow due to the different mechanisms used by *F. hepatica* to modulate the host immune response, which have rendered attempted therapies ineffective in killing the parasite [7–9]. One of these mechanisms is the impairment of the maturation of dendritic cells (DC), which facilitate both parasite survival and modulation of the immune response [10–12].

DC are specialized antigen-presenting cells that can be found in the paracortical area of the lymph nodes where they promote the activation of naive T lymphocytes. On the other hand, follicular dendritic cells (FDC) are cells of stromal origin located in the central region of primary follicles and in the light zone of germinal centres of secondary and tertiary lymphoid organs. They play a crucial role in B-cell activation and have the unique capacity to bind and retain native antigen in B-cell follicles for long periods of time [13, 14]. In previous papers, it has been reported that *F. hepatica* is able to downregulate the Th1 immune response and upregulate the Th2 response at early stages of infection in sheep [15] and mice [16], as well as in chronic stages in cattle [17]. This imbalance towards a Th2 immune profile is mediated through regulatory cytokines and cells, such as DC, that modulate and/or suppress inflammatory responses. It has been determined that different antigenic preparations of this parasite, such as total extract, *F. hepatica* tegumental antigen (FhTeg) and excretory-secretory products (ESPs), decrease the activation state of DC in mice [11, 12, 18, 19], and *F. gigantica* ESPs induce the modulation of buffalo DC [20]. More specifically, it has been reported that FhTeg induces DC modulation, resulting in a lack of T cell Th1 cytokine response and proliferation [21]. In addition, the glycan products produced by *F. hepatica* participate in the modulation of DC maturation and mediate the production of IL-10 and IL-4 during infection, inducing a Th2/regulatory-polarized immune response [22–25]. *F. hepatica* can also induce the development of phenotypes that are characterized by a decreased production of pro-inflammatory cytokines, as well as the expression of general markers that are characteristic of an M2 macrophage phenotype and have been shown to promote the differentiation of Th2 and Treg cells [26–28]. Moreover, it has been reported that *F. hepatica* cathepsin L1 (FhCL1), glutathione transferase (FhGST) and Kunitz-type molecule induce a modulatory effect on DC, which leads to the suppression of the adaptive immune response, including Th1- and/or Th17-related responses [10, 29]. To date, the effect of *F. hepatica* infection on FDC has not been evaluated.

On the other hand, it has been shown that recombinant forms of FhCL1 and FhGST can partially activate DC [10]. Moreover, a mucin-like peptide from *F. hepatica* (Fhmuc) induces parasite-specific adaptive immunity with increased levels of IFN-γ and specific IgG antibodies [30]. In a recent study, it was also reported that the interactions of DC with *F. hepatica* cathepsin L3 (FhCL3) confer a unique expression pattern of the cytokines IFN-γ and IL-13, which may be protective in this parasitosis or in other helminth infections [31].

Most studies investigating the modulation of DC by *F. hepatica* have been carried out in murine models, with only one study in buffaloes [20]. Therefore, to date, there is little available information on *F. hepatica*-induced modulation of DC in ruminants. The aim of the present study was to evaluate the expression of different markers of DC (CD1b, CD83 and MHC-II) and of FDC (CNA.42, S100 and CD83) in the HLNs and liver of sheep during the early stages of infection with *F. hepatica*. These markers have been selected because they have been used as markers of DC, FDC and antigen-presenting cells in formalin-fixed peripheral lymph nodes in sheep [13, 32]. This is the first study to evaluate the immunophenotype of DC and FDC present within the HLNs and livers of sheep experimentally infected with *F. hepatica* in the early stages of infection.

## Materials and methods

### Experimental design

Fifteen eight-month-old male Merino-breed sheep obtained from a liver fluke-free farm were used for this study. All animals were tested monthly for parasite eggs by faecal sedimentation, with negative results in all cases. Moreover, prior to challenge, all animals were tested for serum IgG specific for FhCL1 by an enzyme-linked immunosorbent assay (ELISA), with negative results in all cases. Animals were housed indoors (100 m^2^ covered and 100 m^2^ uncovered facility) and fed with hay and pellets and given water ad libitum. The study consisted of three groups with five sheep each (*n* = 5), including two infected groups and an uninfected control (UC) group. Sheep were orally infected with one dose of 150 metacercariae of the Italian strain of *F. hepatica* (Ridgeway Research Ltd, UK), and all animals were euthanised in batches of five according to the groups at 9 and 18 days post-infection (dpi), being the UC group euthanised at the end of the trial. Sheep did not receive vaccines or adjuvants. In all animals, euthanasia was conducted by intravenous injection of 7 mL of embutramide (200 mg) and mebezonium iodide (50 mg). No adverse reactions or clinical signs were noted during the experiments. The experiment was approved by the Bioethics Committee of the University of Cordoba (code No. 1118) and conducted in accordance with European (2010/63/UE) and Spanish (RD 1201/2005) directives on animal experimentation.

### Histopathology

At necropsy, the liver was removed, and the visceral and diaphragmatic aspects were photographed for gross evaluation. Liver and HLN tissue samples were collected and fixed in 10% neutral buffered formalin for 24 h, then routinely processed and embedded in paraffin wax. Tissue sections (4 µm thick) were stained with haematoxylin and eosin (H&E) for histopathology.

### Immunohistochemical analysis

An immunohistochemical study was used to assess CNA.42, CD1b, S100, MHC-II and CD83 expression in HLN and liver tissue samples using the avidin–biotin–peroxidase method, as described elsewhere [33]. Briefly, after hydration of samples, antigen retrieval was carried out using different methods depending on the antibody. The anti-CNA.42 treatment consisted of incubating the slides with citric acid (pH 9), followed by heating in a microwave for 20 min. anti-MHC-II was incubated with citric acid (pH 6), followed by heating in a microwave for 10 min. anti-CD83 and anti-CD1b were subjected to pronase digestion, and anti-S100 did not require any specific treatment. The samples were rinsed twice for 5 min in phosphate-buffered saline (PBS) and 5 min in PBS-Tween 80 (Panreac, Barcelona, Spain). Endogenous peroxidase activity was blocked by incubation with 3% hydrogen peroxide (Panreac, Barcelona, Spain) in methanol (Panreac, Barcelona, Spain). The slides were then rinsed twice for 10 min in PBS and incubated with 25% normal goat serum (Vector Laboratories, Burlingame, California, USA) for 30 min at room temperature. Each primary antibody was diluted 1:100 in PBS containing 10% normal goat serum, applied to the slides and incubated overnight at 4 °C. The primary antibody details are shown in Table 1. We then rinsed the slides three times in PBS prior to the addition of secondary antibody. Biotinylated goat anti-rabbit immunoglobulin serum (Dako-Agilent, Santa Clara, California, USA), was diluted 1:200 and applied to the slides incubated with the primary polyclonal S100 antibody, whereas biotinylated goat anti-mouse immunoglobulin serum (Dako-Agilent, Santa Clara, California, USA), diluted 1:50, was used for the slides incubated with the primary monoclonal CNA.42, MHC-II, CD83 and CD1b antibodies. Slides with secondary antibodies were incubated for 30 min at room temperature. After three rinses for 10 min in PBS, an avidin–biotin–peroxidase complex (Vector Laboratories, Burlingame, California, USA) diluted 1:50 in PBS was applied for 1 h at room temperature in darkness. The tissue sections were washed three times in Tris-buffered saline (pH 7.2) and incubated with the vector NovaRED^®^ peroxidase substrate (Vector Laboratories, Burlingame, California, USA) for 2 min. Then, samples were rinsed in tap water, lightly counterstained with Mayer’s haematoxylin and mounted with Eukitt^®^ (Freiburg, Germany). Specific primary antibodies were substituted with PBS or non-immune isotype-matched sera as negative controls. HLN sections from sheep were used as positive controls.

**Table 1:** **Specifications of the primary antibodies used for immunohistochemistry**

Specificity	Type	Clone/product code	Dilution	Source
FDC	Mouse anti-human mAb^a^	CNA.42/M7157	1:10	Dako
MHC-II	Mouse anti-human mAb^b^	HI2A/M0748	1:100	VMRD
CD83	Mouse anti-human mAb^a^	HB15e/MCA1582GA	1:50	BioRad
CD1b	Mouse anti-bovine mAb^c^	CC20/MCA2058G	1:10	BioRad
S100	Rabbit pAb^a^	-/Z0311	1:200	Dako

mAb: monoclonal antibody, pAb: polyclonal antibody.

^a^Antibody with cross-reactivity proved in sheep [13].

^b^Antibody with cross-reactivity proved in sheep [66].

^c^Antibody with cross-reactivity proved in sheep (manufacturer data sheet).

### Morphology and cell counting

**Figure 1:**
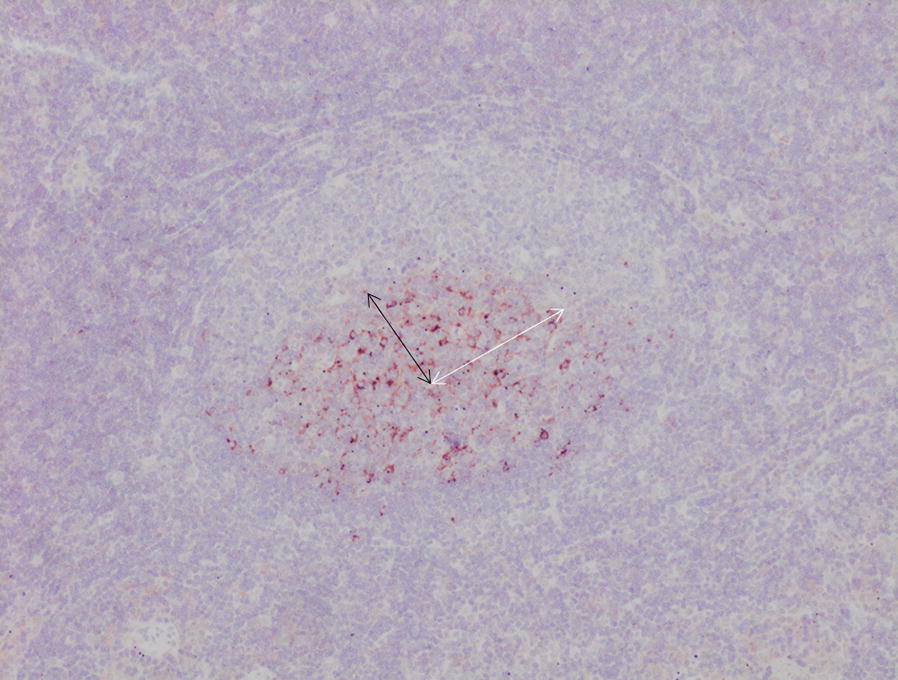
**CNA.42+ cells in a germinal centre inside a lymph node follicle from a sheep 18** **dpi.** The major radius (white arrow) and the minor radius (black arrow) are marked. ABC-haematoxylin counterstain, ×100.

**Figure 2:**
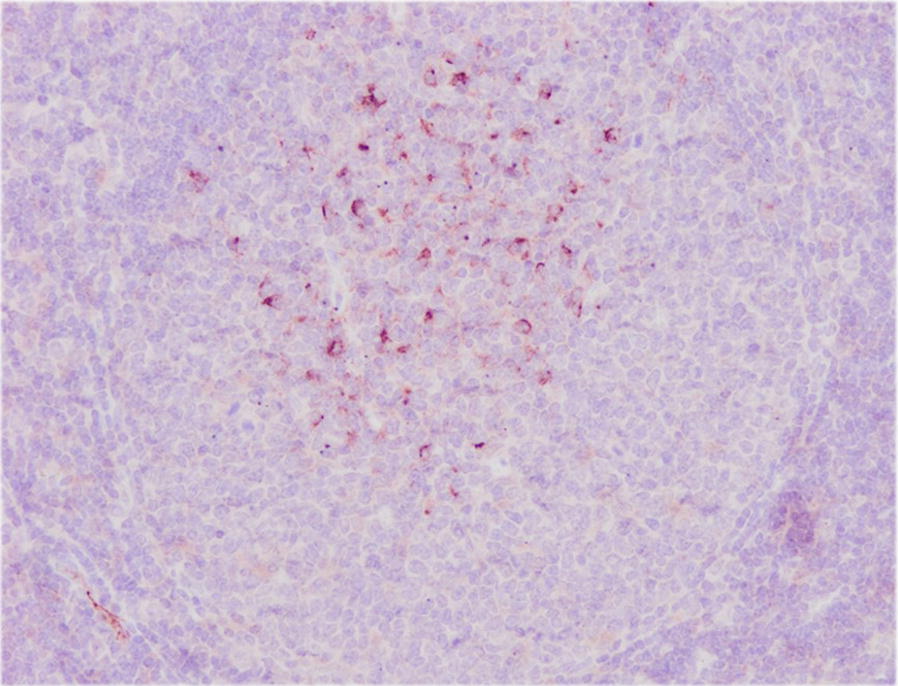
**CNA.42+ cells in a germinal centre in follicles of a lymph node from a sheep 18** **dpi.** ABC-haematoxylin counterstain, ×200.

**Figure 3:**
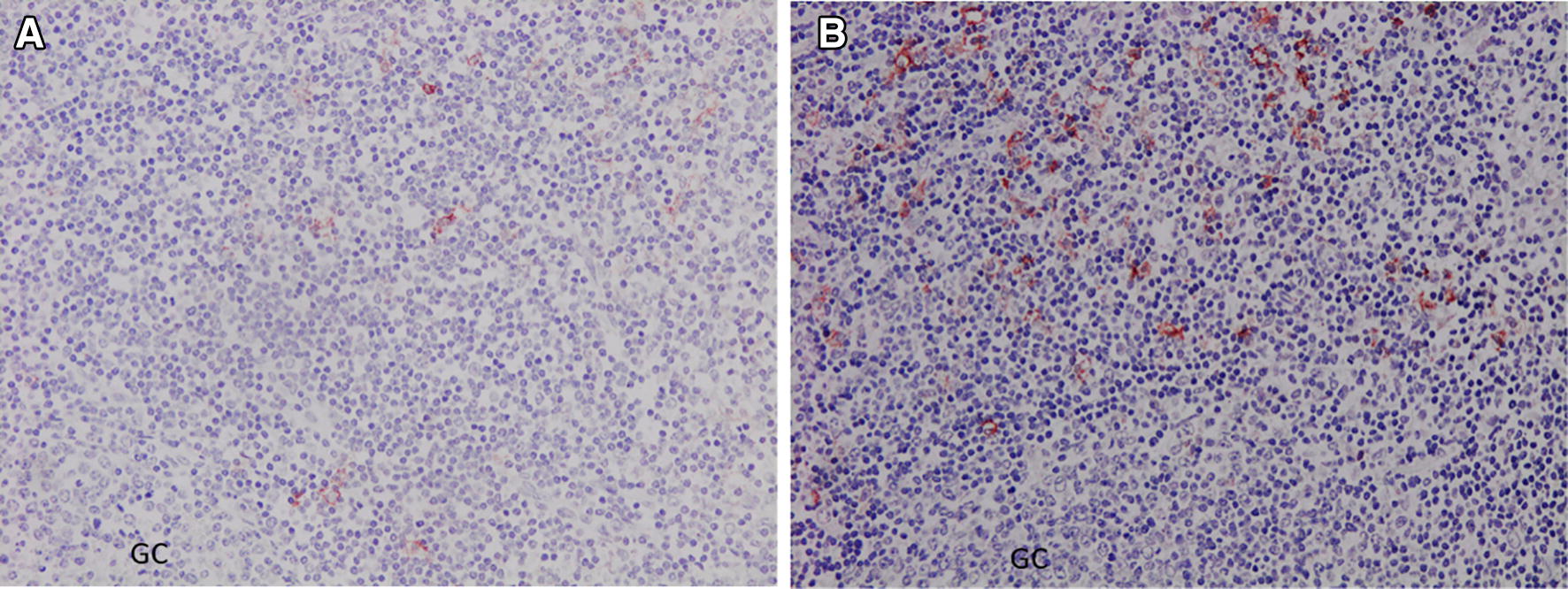
**CD1b+ cells in the paracortical area of a lymph node. A**. From an uninfected control sheep. **B.** From a sheep 18 dpi. GC: germinal centre. ABC-haematoxylin counterstain, ×200.

**Figure 4:**
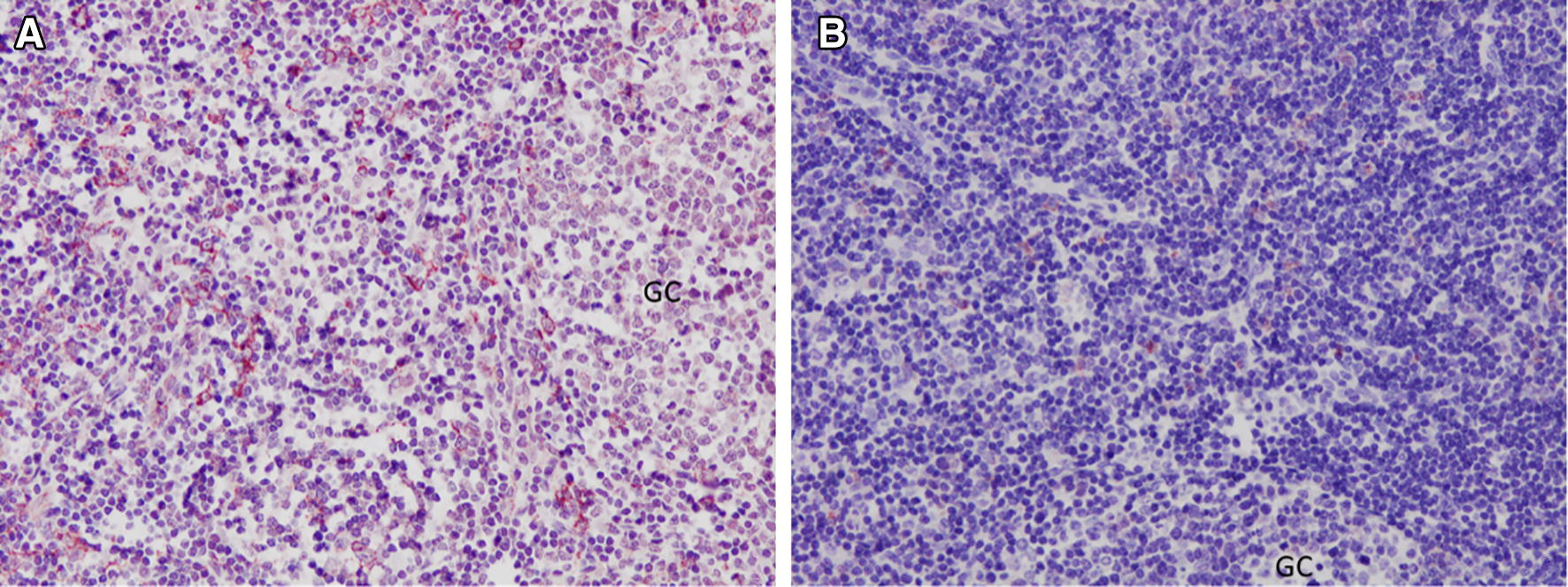
**MHC-II+ cells in the follicular and paracortical area of a lymph node. A**. From an uninfected control sheep. **B.** From a sheep 18 dpi. GC: germinal centre. ABC-haematoxylin counterstain, ×200.

**Figure 5:**
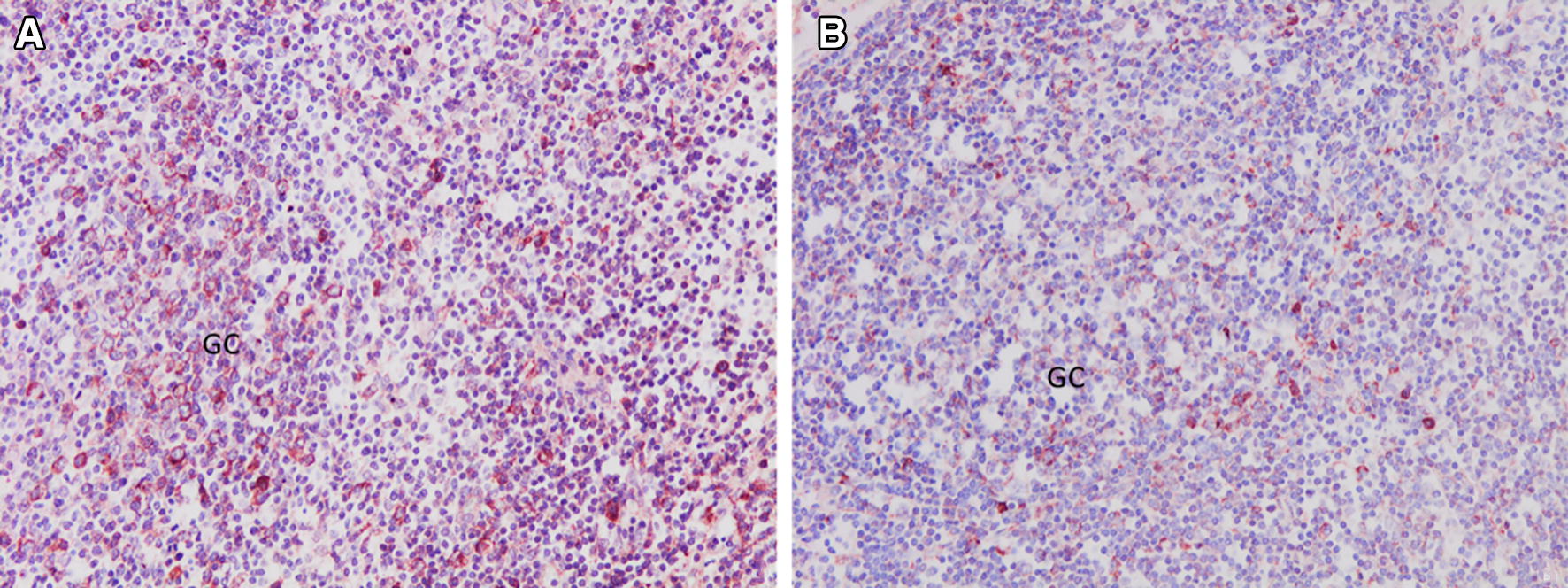
**CD83+ cells in the follicular and paracortical area of a lymph node. A**. From an uninfected control sheep. **B.** From a sheep 18 dpi. GC: germinal centre. ABC-haematoxylin counterstain, ×200.

A morphological analysis was carried out to evaluate the size of CNA.42+ germinal centres in infected and control animals. The length of the major and minor radii of five random follicular germinal centres was measured, and the area (expressed in µm^2^) was calculated as follows: π × a × b, where a was the minor radius, and b was the major radius (Figure 1). Ten random microphotographs of 0.08 mm^2^ were taken, and five follicular germinal centres per animal were measured and multiplied to obtain the area expressed in µm^2^. This calculation, together with the cell count of CNA.42+, CD1b+, S100+, MHC-II+ and CD83+ cells in the HLN and liver tissue sections, was performed using the biomedical software ImageJ v.1.51d. One lymph node and one liver slide per animal were used, five random microphotographs at ×400 magnification were taken from each slide, and the number of positive cells was counted. Specifically, CNA.42+ cells were counted inside lymph node follicular germinal centres (Figure 2); MHC-II+ (Figure 4), CD83+ (Figure 5) and S100+ cells were counted in the follicles, paracortical and medullary areas; and CD1b was counted in the paracortical and medullary areas (Figure 3). The results are expressed as the mean ± SD per group.

### Statistical analysis

Statistical analysis was carried out using the GraphPad Prism 7.0 software package (GraphPad Software, Inc., San Diego, USA). The Kolmogorov–Smirnov test was applied to evaluate whether distributions were parametric. Comparisons between groups were made using the Mann–Whitney test for non-parametric distributions. Correlation studies were carried out using the Spearman correlation test for nonparametric distributions. *P* < 0.05 was considered significant.

## Results

### Histopathology

Livers from the UC group showed no histopathological changes. Portal spaces showed occasional lymphocytes, and eosinophils were not found in the negative control livers. All animals at 9 dpi presented necrotic foci and focal haemorrhages, mainly involving the subcapsular areas. Necrotic foci showed abundant cell debris and a mild to moderate infiltrate of eosinophils, often with pyknotic nuclei. Necrotic tracts started 0.5–1.5 mm behind migrating larvae. Animals at 18 dpi presented microscopic lesions characterized by the presence of necrotic foci and tortuous pathways located in the hepatic parenchyma, mainly in the subcapsular areas. Associated with the foci and necrotic paths, we found a variable inflammatory infiltrate composed mainly of eosinophils, macrophages and occasional neutrophils, together with cellular debris and peripheral haemorrhages. The portal spaces adjacent to the necrotic foci showed a severe inflammatory infiltrate, composed mainly of lymphocytes, macrophages and eosinophils. These findings suggest that the pathway of entry of inflammatory cells into the hepatic parenchyma is through the portal veins. At 18 dpi, granulomas were also found, and they were composed of a necrotic centre surrounded peripherally by macrophages arranged in palisade and lymphocytes and eosinophils.

The HLNs of the UC group showed a small cortex with primary lymphoid follicles or secondary lymphoid follicles with small germinal centres. Paracortical areas and medullary cords were also small and composed of lymphocytes, macrophages and occasional plasma cells. Eosinophils were not found. The area of germinal centres was 23 953 ± 5380 µm^2^ in the UC group and 47 585 ± 4321 µm^2^ at 9 dpi and 61 631 ± 4743 µm^2^ at 18 dpi in the infected groups. Significant differences were found between the UC group and the infected group 9 and 18 dpi (*P* < 0.01 and *P* < 0.001, respectively). The medullary cords also showed hyperplasia at 9 and 18 dpi due to increased numbers of lymphocytes, plasma cells and macrophages. Infiltration of eosinophils was variable in the paracortical area and medulla of infected animals, in which hemosiderin pigment was found in the cytoplasm of a variable number of medullary macrophages.

### Immunohistochemistry

The anti-CNA.42 mAb yielded cytoplasmic immunostaining in FDC, mainly located in germinal centres of the secondary lymphoid follicles of HLNs (Figures 1 and 2). Only FDC located in the lymphoid follicles were counted. The results of the immunohistochemical study of CNA.42+ cells are summarized in Table 2. The number of CNA.42+ FDC in the germinal centres of the HLN follicles in animals at 18 dpi increased significantly (*P* < 0.001) in comparison with the that in the UC group. In the liver, CNA.42+ cells showed stellate shape and they were observed in the inflammatory infiltrates composed of numerous lymphoid cells arranged in lymphoid follicles surrounding portal spaces and in the periphery of granulomas. The number of CNA.42+ FDC in the liver increased significantly at 9 dpi (*P* < 0.05) and 18 dpi (*P* < 0.01) compared to that in the UC group (Table 2). However, high individual variability was observed in the liver for CNA.42, in part due to the variability in the extent of the inflammatory infiltrates arranged in lymphoid follicles. CNA.42+ cells were not found in the UC group.

**Table 2:** **Number of CD1b+, S100+, MHC-II+ and CD83+ cells in HLNs (medullary, paracortical and follicular areas) and livers expressed as the mean ± SD per area of 0.08** **mm**^**2**^**per animal**

Antibody	Group	Medullary area HLNs	Paracortical area HLNs	Follicular area HLNs	Livers
CNA.42	UC	–	–	43.1 ± 11	–
	9 dpi	–	–	71.6 ± 6.3	6.5 ± 10.8
	18 dpi	–	–	93.2^c^ ± 10.1	6.8 ± 10.1
CD1b	UC	6.2 ± 0.8		–	–
	9 dpi	12.7 ± 2.2		–	2.1 ± 3.4
	18 dpi	19.6^c^ ± 4.6		–	5.7^b^ ± 3.6
S100	UC	8.7 ± 2.6	9.0 ± 6.9	14.9 ± 10.1	1 ± 0.1
	9 dpi	4.7 ± 0.9	5.8 ± 2.0	6.8 ± 4.3	2.3 ± 1.5
	18 dpi	12.6^b^ ± 1.5	16.3^a^ ± 4.6	21.8^a^ ± 7.1	3.9^b^ ± 2.5
MHC-II	UC	19.8 ± 1.7	34.2 ± 1.5	16.1 ± 1.7	11.8 ± 9
	9 dpi	13.4^a^ ± 3.1	16.2^a^ ± 3.8	1.6^d^ ± 0.8	23.6^b^ ± 5.5
	18 dpi	1.9^d^ ± 0.6	7.7^d^ ± 4.2	0.2^b^ ± 0.7	34^d^ ± 9
CD83	UC	26.8 ± 6.4	53.1 ± 13.6	84 ± 10.4	0.6 ± 0.6
	9 dpi	67.4^b^ ± 10.7	27^a^ ± 5.4	29.7^a^ ± 9.4	2 ± 2.9
	18 dpi	75^b^ ± 9.8	34.5 ± 1.4	18.9^a^ ± 2	4.9^c^ ± 2.5

CNA.42 is expressed as the number of positive cells per germinal centre. Statistical differences are in comparison with the UC group.

UC: uninfected control group, dpi: days post-infection.

^a^(*P* < 0.05), ^b^(*P* < 0.01), ^c^(*P* < 0.001), ^d^(*P* < 0.0001).

The anti-CD1b mAb yielded cytoplasmic immunostaining in DC located in paracortical and medullary areas of HLNs (Figure 3). The results of the immunohistochemical study of CD1b are summarized in Table 2. The number of CD1b+ DC in HLNs at 18 dpi increased significantly (*P* < 0.0001) compared to that in the UC group. In the liver, CD1b+ cells were observed in the inflammatory infiltrates surrounding portal spaces and in the periphery of granulomas. The number of CD1b+ DC in the liver increased significantly at 18 dpi (*P* < 0.01) compared with that in the UC group (Table 2).

The anti-S100 pAb yielded nuclear and/or cytoplasmic immunostaining in peripheral nerves (internal positive control). In addition, only stromal stellate cells located in lymphoid follicles, paracortical areas and medullary cords of HLNs were considered for the cell count. The results of the immunohistochemical study of S100 are summarized in Table 2. The number of S100+ stromal cells in lymphoid follicles and in paracortical and medullary areas in HLNs showed no significant differences between infected animals (9 and 18 dpi) and uninfected controls. In the liver, S100+ cells were observed in inflammatory infiltrates surrounding portal spaces and in the periphery of granulomas. The number of S100+ stromal cells in the liver increased significantly at 18 dpi (*P* < 0.01) in comparison with the number in the UC group.

The anti-MHC-II mAb yielded cytoplasmic immunostaining of stellate cells located in follicles, paracortical areas and medullary areas of HLNs (Figure 4). A variable number of lymphocytes showed a positive MHC-II immunoreactivity; however, only cells with large cytoplasm and cytoplasmic prolongations, an appearance compatible with DC, were considered for the cell count. The results of the immunohistochemical study of MHC-II are summarized in Table 2. The number of MHC-II+ cells decreased significantly in HLN lymphoid follicles, paracortical and medullary areas at 9 and 18 dpi compared to that in the UC group. In the liver, MHC-II+ cells were found in the inflammatory infiltrates of portal spaces and in the periphery of granulomas. The number of MHC-II+ cells in the liver increased significantly at 9 dpi (*P *< 0.01) and at 18 dpi (*P *< 0.0001) in comparison with that in the UC group (Table 2).

The anti-CD83 mAb yielded cytoplasmic immunostaining in stellate cells located in lymphoid follicles, paracortical areas and medullary cords of HLNs (Figure 5). The results of the immunohistochemical study of CD83 are summarized in Table 2. The number of CD83+ FDC decreased significantly in lymphoid follicles at 9 dpi (*P* < 0.05) and at 18 dpi (*P* < 0.05), as well as the number of CD83+ DC in the paracortical areas at 9 dpi (*P* < 0.05) and mononuclear cells in the medulla at 9 dpi (*P* < 0.01) and at 18 dpi (*P* < 0.01), in comparison with the respective numbers in the UC group. In the liver, CD83+ mononuclear cells were found in the inflammatory infiltrates of portal spaces and in the periphery of granulomas. Since the livers of the UC group showed only occasional inflammatory cells (lymphocytes, macrophages and stellate cells) in the portal areas, the number of CD83+ cells was quite low. In the infected groups, the number of CD83+ cells in the liver increased significantly at 18 dpi (*P* < 0.001) in comparison with that in the UC group (Table 2).

## Discussion

The gross pathology and microscopic changes (acute necrotic foci and tracts behind migrating larvae) found in the liver at 9 and 18 dpi are in accordance with the gross lesions reported during early stages of infection in sheep [34, 35] and goats [36, 37]. The marked hyperplasia of HLN lymphoid follicles and medullary cords found at 9 and, particularly, at 18 dpi suggests a strong local humoral immune response, as reported previously in sheep with chronic fasciolosis [34, 38–40].

The CNA.42 antibody was expressed mainly by FDC located in the light zones of the germinal centres of lymphoid follicles, an expression pattern that was also reported for human [41] and sheep [13] lymph nodes using the same antibody. Sheep FDC have been identified as cells forming a reticular pattern in the light zone of the germinal centres and playing important roles in the selection of memory B lymphocytes during germinal centre reactions [13, 42].

The significant increase in the expression of the CNA.42 antibody at 18 dpi, compared to that in the UC group, coincided with marked hyperplasia of germinal centres at 18 dpi. This result agrees with the marked increased expression of the same antibody in human hyperplastic germinal centres reported in other conditions [41]. The increased expression of CNA.42 in the liver at 18 dpi may be related to the substantial increase in inflammatory infiltrate at 18 dpi compared to that seen in the UC group, in which only occasional inflammatory cells were found in the portal spaces. The present study is the first to analyse the expression of CNA.42 in *F. hepatica*-infected ruminants, and there are a limited number of published studies describing the expression of FDC in other helminth infections. It has been reported that the enteric nematode *Heligmosomoides polygyrus* induces FDC overexpression in mouse mesenteric lymph nodes [43, 44]. Similarly, subcutaneous reactive lymph nodes from human patients suffering onchocerciasis showed strong expression of FDC in germinal centres [45]. These results agree with the increased number of CNA.42+ cells in both the liver and HLNs of *F. hepatica*-infected sheep found at 18 dpi in the present study.

Previous studies have reported the presence of a second population of FDC, localized to the dark zones of sheep germinal centres, but their function has yet to be defined [13, 46–48]. These cells did not express the classical FDC marker CNA.42 but stained positively for CD83 and S100, suggesting that the FDC in the dark zone are a less specialized subpopulation of reticular cells that might share a similar origin with the light zone FDC [13]. The number of S100+ stromal cells increased in HLN follicles and paracortical and medullary areas of animals at 18 dpi, compared with that seen in animals at 9 dpi. In the liver, the number of S100+ stromal cells increased significantly at 18 dpi in comparison with that in the UC group.

Furthermore, the S100 protein is expressed by activated FDC, which may explain why anti-S100 gave rise to individual cell staining. Transcriptomic analysis of PBMCs from sheep revealed an upregulation of S100 genes at 2 weeks post-infection, and these cells were shown to be involved in leukocyte migration and the innate immune response [49]. In a murine model, the S100A8 gene was also overexpressed in *F. hepatica* infections [50].

The CD1b molecule is expressed by a mature population of migrating DC from sheep peripheral lymph nodes and induces the proliferative response of CD4+ T cells and antigen presentation to promote pro-inflammatory (IL-6), pro-Th1 (IL-12p40) and anti-inflammatory (IL-10) responses. These responses were amplified by *Salmonella* antigens and limited to only IL-6 induction by helminth secretions [32, 51]. *Leishmania* spp. infection of human monocyte-derived dendritic cells resulted in reduced expression of CD1b [52]. Similar results were found in bovine monocyte-derived macrophages infected with *Neospora caninum* [53]. These results contrast with those found in the present study, in which the number of CD1b+ DC increased significantly in HLNs and livers at 18 dpi compared to that in the UC group. The difference in the expression of CD1b in the two parasites may be related to differences in the host response to *N. caninum* and *F. hepatica* infections in cattle and sheep, respectively. Thus, in *N. caninum*-infected cattle, elevated reactive oxygen species (ROS), IL-10 and IFN-γ production has been reported [53], whereas in *F. hepatica*-infected sheep, decreased ROS production [54] and decreased IFN-γ gene expression have been observed [15].

The results of the present study reveal a significant decrease in the cellular expression of the antigen presentation markers CD83 and MHC-II by DC and FDC in the lymph nodes of sheep experimentally infected with *F. hepatica*. CD83 is broadly used as a maturation marker for human and mouse DC [55, 56]. Nevertheless, it is also expressed on a variety of different cells, including monocytes and macrophages [56] and activated B and T lymphocytes [57–59]. CD83 expression in sheep has been described in pseudo-afferent lymph DC [60]. The same monoclonal CD83 antibody used in the present work identified DC in the paracortical area of lymph nodes displaying a marked cytoplasmic localization, while it was not detected in macrophages located along the medullary sinuses, and it labelled B cells located in the lymphoid follicles that formed a fine reticulum—a staining pattern consistent with that expected for FDC [13]. In our study, the decreased expression of CD83 in DC and FDC of paracortical areas and germinal centres, respectively at 9 and 18 dpi agrees with the decreased expression of the same cell marker in peritoneal leucocytes from *F. hepatica*-infected sheep at 3 and 9 dpi [54] and with the decrease in the expression of CD83 in monocyte-derived DC cocultured in the presence *Anisakis pegreffi* live larvae and their crude extracts [61], as well as *T. multiceps* [62] and *T. spiralis* [63]. Since the expression of the CD83 molecule in mature DC could have a specialized function during antigenic presentation, contributing to lymphoid activation [64], reduced CD83 expression during *F. hepatica* infection in sheep suggests a modulatory effect that impairs antigenic presentation and, therefore, the host immune response during the early stages of infection. The lower level of CD83 expression in stellate cells of the dark zone of lymphoid follicles observed in the present study agrees with previous studies using the same antibody in sheep lymph nodes [13].

It has been reported that in fixed sheep lymph nodes, MHC-II is expressed at high levels by interdigitating cells present in the paracortex, at lower levels by the B lymphocytes present in the follicles, and at a lower level in macrophages from the medullary sinuses [13], an expression pattern also observed in our study. In the present study, the reduced MHC-II+ DC found in HLNs at 9 and 18 dpi, compared to the number in the UC group, agrees with the significant decrease in MHC-II in the peritoneal leukocytes of sheep experimentally infected with *F. hepatica* at 9 dpi [54], with the reduced expression of MHC-II in DC from buffalos cultured in the presence of ESPs from *F. gigantic*a [20], and with the low MHC-II expression in DC of mice cultured in the presence of mucin-like peptide [30, 65] or in the presence of FhTeg from *F. hepatica* [18].

In summary, an increase in CNA.42+ FDC and CD1b+ DC was found at 18 dpi in HLNs compared with the respective numbers in the UC group, coinciding with marked hyperplasia of the germinal centres of HLNs. However, the expression of MHC-II and CD83 decreased at 9 and, particularly, at 18 dpi in HLNs compared with that in the UC group, suggesting that *F. hepatica* infection induces suppression of markers related to antigenic presentation.

## Data Availability

All data are available, upon reasonable request, from the corresponding author.

## Abbreviations

DC: dendritic cells
FDC: follicular dendritic cells
CD1b: cluster of differentiation 1b
MHC-II: Major histocompatibility complex-II
CD83: cluster of differentiation 83
HLNs: hepatic lymph nodes
UC: uninfected control
FhTeg: *Fasciola hepatica* tegumental antigen
ESPs: excretory secretory products
IL-10: interleukin 10
IL-4: interleukin 4
FhCL1: *Fasciola hepatica* cathepsin L1
FhGST: *Fasciola hepatica* glutathione transferase
Fhmuc: *Fasciola hepatica* mucin-like peptide
IFN-γ: interferon gamma
FhCL3: *Fasciola hepatica* cathepsin L3
IL-13: interleukin 13
ELISA: Enzyme-linked immunosorbent assay
PBS: phosphate-buffered saline
PBMCs: peripheral blood mononuclear cells
IL-6: interleukin 6
IL-12p40: interleukin 12p40
ROS: reactive oxygen species
